# Whole-Genome Sequence of Psychromonas antarctica Strain DSM 10704

**DOI:** 10.1128/mra.00141-22

**Published:** 2022-04-25

**Authors:** Shanmukh Chandaluri, Jonathan C. Thomas

**Affiliations:** a School of Science and Technology, Nottingham Trent University, Nottingham, United Kingdom; Montana State University

## Abstract

Here, we report the first draft genome sequence of the psychrophilic species Psychromonas antarctica. The genome of strain DSM 10704 was sequenced using an Illumina MiSeq instrument; it was assembled into 300 contigs and totaled 3,916,717 bp, with 95× coverage.

## ANNOUNCEMENT

Psychromonas antarctica is a psychrophilic Gram-negative bacterium and was first isolated from pond sediment from the McMurdo Ice Shelf, Antarctica (1). *P. antarctica* is able to survive in extreme climatic conditions, in temperatures between 2 and 22°C and at high salt concentrations (1). However, previously no whole-genome sequence was available for this species.

A freeze-dried stock previously prepared by a former lab member was used to inoculate and grow the strain in marine broth 2216 (BD Difco, UK) at 12°C for 5 days under anaerobic conditions, using a cell culture flask with an Oxoid AnaeroGen sachet in an Oxoid AnaeroJar (Thermo Fisher Scientific, UK). Genomic DNA was extracted using a DNeasy kit (Qiagen) according to the manufacturer’s instructions. Double-stranded DNA (dsDNA) was quantified using a Qubit fluorometer (Thermo Fisher Scientific). A sequencing library was prepared using a Nextera XT DNA sample preparation kit (Illumina) and sequenced on an Illumina MiSeq instrument with 2 × 250-bp paired-end v2 chemistry, according to the manufacturer’s protocol. The quality of the resulting 828,674 paired-end reads was visualized using FastQC v0.11.8 (https://www.bioinformatics.babraham.ac.uk/projects/fastqc/), before the adapters were trimmed using Trimmomatic v0.39 (2). Duplicate reads were removed using clumpify.sh (3), and the reads were quality trimmed using Sickle v1.33 (Phred quality cutoff, Q20; minimum length, 150 bp) (4). The resulting 722,468 paired-end reads were *de novo* assembled using SPAdes v3.14.1 (5). Contigs shorter than 500 bp were discarded, and genome annotation was performed using NCBI’s Prokaryotic Genome Annotation Pipeline (6).

The final assembly consisted of 300 contigs, resulting in 95× coverage of a 3,916,717-bp draft genome, with an *N*_50_ value of 32,057 bp and a GC content of 39.98%. Annotation predicted 3,437 coding DNA sequences (CDSs), including 60 tRNAs, 11 rRNAs, and 4 noncoding RNAs (ncRNAs), as well as 75 pseudogenes.

### Data availability.

All data are available under BioProject accession number PRJNA739283. The draft whole-genome sequence of Psychromonas antarctica strain DSM 10704 was deposited in GenBank under accession number JAHNZV000000000. The version described in this paper is version JAHNZV000000000.1. The Illumina raw read data were submitted to the SRA and are available via accession number SRR14867394.
